# Nursing Protagonism in the UN Goals for the people’s health

**DOI:** 10.1590/1518-8345.0000.2864

**Published:** 2017-01-30

**Authors:** Isabel Amélia Costa Mendes, Carla Aparecida Arena Ventura

**Affiliations:** 1 Full Senior Professor, Escola de Enfermagem de Ribeirão Preto, Universidade de São Paulo, PAHO/WHO Collaborating Centre for Nursing Research Development, Ribeirão Preto, SP, Brazil. E-mail: iamendes@usp.br; 2 Associate Professor, Escola de Enfermagem de Ribeirão Preto, Universidade de São Paulo, Centro Colaborador da OPAS/OMS para o Desenvolvimento da Pesquisa em Enfermagem, Ribeirão Preto, SP, Brasil. E-mail: caaventu@eerp.usp.br

**Figure:**
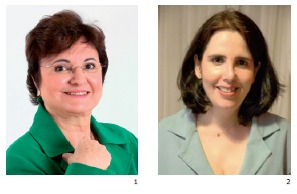

For decades, high-level discussions on possible actions and strategic goals have been conducted aiming at achieving a better level of health for the people, involving government representations of member countries, civil society, private sector and academic leaders under the World Health Organization (WHO) leadership and, with the participation of other agencies of the United Nations Organization (UN). In this sense, principles for the organization of national health services and systems, considering human resources training for health, have been discussed since the Alma-Ata and have materialized in specific objectives and indicators through the Millennium Development Goals (MDGs), and now with the Sustainable Development Goals (SDG). Moreover, despite cooperation efforts and investments in infrastructure and human resources training, it was still not possible to align results with projected expectations. In order to target health investment to achieve these goals, the WHO has produced a practical guide to adapt funding for the provision of universal health coverage^1^.

In the imminence of the milestone established for the achievement of the Millennium goals, based on a broad consultation, in 2012, leaders listed positive results derived from this policy with regard to the objectives related to the health area, the main ones are: increased health funding, more progress since 2000 than in previous periods; influence of political discourse at the highest levels, with indicators driven by concrete quantifiable goals and objectives. On the other hand, weaknesses were clear: lack of focus on equity and human rights as well as instabilities in the process, contributing to accentuate the fragmented health approach. Given this context, the goals for the post 2015 period were: to maintain as a priority the millennium goals related to health; attention and measures to refrain risk factors and promote care to the population with non-communicable diseases; equity and human rights as fundamental principles in all contexts; strengthening health systems, now with more attention to specific factors such as mental health, nutrition, adolescence, sexual and reproductive health^2^. Based on this consultation, consensuses were produced focusing on the assumption that health acts both as a trigger and as a beneficiary of the development of other society sectors, giving further evidence to social determinants of health. Among other consensuses derived from this process, health stands out as a human right and the goal is that the post 2015 health development agenda be allocated to all countries:- therefore, universality is the scope.

The definition of post 2015 health goals was thus defined: maximizing healthy life, through access and universal health coverage, which presupposes an increase in essential services coverage; equity with financial protection and strengthening of health systems through qualified personnel. In defining the goals to be achieved, the ways in which they can be attained emerged; it is evident that and equity must be introduced in all the goals; that investment in health systems implies systematically promoting the training and development of human resources competences, and also generating information systems capable of analysis and use of data; that countries take national responsibility for goals and ensure responsiveness to global commitments in their territory, promoting partnerships for the effective implementation of programs to achieve better results.

One great challenge is the global shortage of healthcare professionals, especially nursing - a profession with 19.3 million nurses in the world, with a global density of 29 nurses and obstetricians for every 10 million people. However, more than 70% of the UN member states report a shortage of these professionals in their territories^3^ ^-^ ^5^, since this shortage is accompanied by poor geographic distribution - inter and intra-country, as well as fragility in the composition of skills, which determines the comprehensive overview of the crucial problem of human resources. Thus, this challenge must be faced very carefully by all countries, each in its own way, using different conceptual nursing models, where ethical aspects and human rights are guided by a vision of access and universal health coverage and involvement in global health initiatives^6^. For that matter, it is not possible to prescind from partnerships that make feasible and ensure the sustainability of the process, since it involves mutual interests, collaboration, institutional commitment from a global health perspective to build bridges, and overcome cultural barriers, aiming the development of capacities.

The adoption of the universal health coverage agenda represents an opportunity to shape the development of the workforce in the area and rationalize the demands of health care, considering that an effective health care system does not exist without adequate personnel to perform it. Thus, the discussions about the insertion of the advanced nursing practice, adding value to nursing human resources and to local health systems, are relevant. The success offer rate of skilled and people-centered service will only be achieved through re-engineering the health workforce, with reforms in education programs that shape the next generation of professionals admitted through a fair process of competitive and progressive recruitment, together with re-valorization of the service they provide^7^.

And, of course, institutions need to once and for all incorporate and embrace the concept that this human capital must have access to continuing education, valued career, and treatment as real assets of the organizations they serve. Balancing the need for growth with the capacity to execute consists of a challenge that must be continuously measured in the light of goals and policies. In order to increase the assistance of increasing demand, training new professionals who are qualified and adapted to the same health policy is imperative. Whether in public or private organizations, the management of academic and health services must have leaders capable of evaluating these dimensions and, pervaded with the same spirit, seek a joint decision. Since in the health area the value creation of the service given to the public is due to performance quality of the professionals, the leaderships should clearly establish quality goals and invest in their teams, which presuppose the interdependence of competences and motivation of the workers. Therefore, in all instances in vocational training and throughout the working life, the premise of skill development and professional valorization is essential when considering human capital as the most valuable asset of service organizations. In the health services sector and in the context of inequalities and imbalances in the workforce, nursing can contribute more significantly, which imposes on schools the more intense training of generalist nurses, but also on nurses qualified for advanced nursing practices, so to meet the needs of health systems and to meet the goal of universal access and coverage at global, regional and national levels. Such a contribution can only be triggered as far as the political system and civil society really do give value to these professionals. Without market demand, there will be no attractive reasons for the younger people to choose this career, even if they have inclination to the area.
